# Clinical Response of Nuberol Forte® for Pain Management With Musculoskeletal Conditions in Routine Pakistani Practice (NFORTE-EFFECT)

**DOI:** 10.7759/cureus.23011

**Published:** 2022-03-09

**Authors:** Syed Shahid Noor, Muhammad Kazim Najjad, Nasir Ahmed, Khurram Anwar, Abdul Memon, Tehseen Riaz, Muhammad Hanif, Nauman Maqbool, Saeed Ahmed, Israr Ahmed, Ali Yasir Khanzada

**Affiliations:** 1 Orthopedics and Trauma, Liaquat National Hospital and Medical College, Karachi, PAK; 2 Orthopedic Surgery, Liaquat National Hospital, Karachi, PAK; 3 Orthopedics, Liaquat University of Medical and Health Sciences, Jamshoro, PAK; 4 Periodontology, Isra University Hospital, Hyderabad, PAK; 5 Family Medicine, Family Clinic, Mirpurkhas, PAK; 6 Orthopedic Surgery, Jinnah Hospital, Lahore, PAK; 7 Orthopedic Surgery, Lahore General Hospital, Lahore, PAK; 8 Orthopedic Surgery, Fauji Foundation, Rawalpindi, PAK; 9 Orthopedics, Life Care International Hospital, Islamabad, PAK; 10 Orthopedics and Spinal Surgery, Hayatabad Medical Complex, Peshawar, PAK; 11 Clinical Research, The Searle Company Limited, Karachi, PAK

**Keywords:** pakistani population, safety, effectiveness, quality of life, pain management, orphenadrine/paracetamol combination, musculoskeletal pain

## Abstract

Background

Musculoskeletal pain is the most common complaint presented to the health practitioner. It is well-known that untreated or under-treated pain can have a significant negative impact on an individual’s quality of life (QoL).

Objectives

The current study aimed to assess the clinical response of Nuberol Forte® (paracetamol 650 mg + orphenadrine 50 mg) to musculoskeletal pain in routine Pakistani practice and its impact on improving the patient’s QoL.

Methods

A prospective, observational multicenter study (NFORT-EFFECT: Safety & Efficacy of Nuberol Forte in Pain Management). Three hundred ninety-nine patients with known prescreened musculoskeletal pain were recruited from 10 major healthcare facilities across six (6) major cities of Pakistan, as per the inclusion/exclusion criteria. After the baseline visit (Visit 1), the patients were followed up one to two weeks (Visit 2) after the treatment as per the physician's discretion. Data were collected using the Case Report Form (CRF) designed for the study, and adverse events (AEs) were also monitored to assess drug safety. Pain intensity was assessed through a visual analog scale (VAS), and QoL was assessed using the Muscle and Joint Measure (MJM) scale.

Results

Out of 399 enrolled patients, 49.4% were males and 50.6% were females with a mean age of 47.24 ± 14.20 years. Most patients were presented with knee osteoarthritis (OA), i.e., 148 (38%), followed by backache 70 (18.2%). A significant reduction in the mean pain score was observed after treatment with the combination of paracetamol and orphenadrine (p<0.05). Furthermore, an overall improvement in the patient’s QoL was also observed. During the study, only 10 patients reported mild adverse events (AEs), namely, dryness of the mouth, dizziness, gastric irritation, tachycardia, restlessness, etc.

Conclusion

The combination of paracetamol and orphenadrine (Nuberol Forte) exhibited effective pain management among patients with musculoskeletal conditions and improved their QoL.

## Introduction

Musculoskeletal conditions are challenging disorders for patients and treating doctors [1]. It is the fourth leading cause of the disease burden, and according to the Global Burden of Disease (GBD), approximately 1.71 billion people of all ages have musculoskeletal conditions worldwide [2]. In the South-East Asian region, 369 million are affected by musculoskeletal conditions [3]. There is a scarcity of data representing disease prevalence in Pakistan; however, multiple studies focused on specific professional communities with high risks to musculoskeletal disorders [4-6]. Musculoskeletal pain is instigated by almost 150 conditions primarily involving the joints, bones, muscles, and spine and other aspects like inflammatory diseases and vasculitis [3-8].

The musculoskeletal pain affects the patient’s quality of life (QoL) and unusually visual analog scale (VAS), a unidimensional measure for assessing acute and chronic pain [9-10]. It is well-known that untreated or under-treated pain can have a significant negative impact on an individual’s daily life [11]. Assessment of health-related quality of life (HRQoL) provides a way for clinicians to better understand the effect of this chronic condition [11-13].

The two main approaches to measuring the QoL includes generic and disease-specific instruments. In generic tools, the short form 36 questionnaire (SF-36) and the Euroqol five questionnaire (EQ-5D) are the most frequently used questionnaires enabling quantification of the HRQoL in people with musculoskeletal disorders [11-13]. However, the generic tools are not capable of detecting the effects of a specific condition on QoL [11-13]. In the current study, the Muscle, Joint Measure (MJM) scale was applied to assess the overall conditions of patients suffering from pain, stiffness, backache, or joint pain [12,14].

From a therapeutic perspective, a multimodal, multidisciplinary approach using fixed-dose combinations of analgesics potentiates pain relief and reduces the consequent risk of adverse events (AEs) [15]. In the light of WHO pain guidelines, nonopioid analgesics, like paracetamol, should be trialed first, followed by non-steroidal anti-inflammatory drugs (NSAIDs) and then opioids with caution, based on pain characteristics [16]. NSAIDs are associated with an increased risk of adverse events like cerebral/cardiovascular events, gastrointestinal complications, the relative risk of acute myocardial infarction, and renal adverse effects [17]. The risks are associated with the dose and the duration [17].

Multiple studies evaluating the clinical response of paracetamol fixed-dose with different combinations (ibuprofen, tramadol, diclofenac, aceclofenac) as an analgesic have been conducted [18-19]. Despite the widespread use of a combination of paracetamol with orphenadrine, no local data are available to evaluate the effectiveness, safety, and tolerability of fixed-dose orphenadrine plus paracetamol in managing musculoskeletal pain [4-6]. Only one publication exists in the past, including the same brand with different strengths and indications [20]. Therefore the current study was conducted to assess the clinical response of Nuberol Forte® (paracetamol 650 mg + orphenadrine 50 mg) for pain management with musculoskeletal conditions in routine Pakistani practice. Through this research, we intended to assess the effectiveness, quality of life, safety, and tolerability of Nuberol Forte among the local Pakistani population.

## Materials and methods

Study design and population

This NFORT-EFFECT observational, prospective multicenter study was conducted in compliance with Good Clinical Practice guidelines and local regulatory requirements. The study sponsor was The Searle Company Limited, Pakistan. To maintain the GCP compliances, the sponsor assigned the CRO for the site and data management.

A total of 399 patients with known, prescreened musculoskeletal conditions and pain who attended the study sites were enrolled. All consenting patients aged ≥18 and ≤70 years, inclusive of either sex, were kept in the inclusion criteria. While patients with known hypersensitivity to the Nuberol Forte product, the metabolites, or formulation excipients, those with glaucoma, prostatic hypertrophy or obstruction at the bladder neck, myasthenia gravis, oesophageal spasm, and pyloric or duodenal obstruction were excluded from the study. Also excluded were pregnant or breastfeeding women and those treated with Nuberol Forte to evaluate safety as per approved prescribing information for Nuberol Forte in Pakistan.

The recruited patients were then prescribed paracetamol (650 mg) and orphenadrine (50 mg) combination (Nuberol Forte) for seven to 14 days as per the investigator's discretion based on the pain intensity. After the initial screening (Visit 1), a follow-up visit was conducted after one to two weeks of the treatment (Visit 2).

Study endpoints

The primary objective was to assess the pain management response of Nuberol Forte treatment and the overall safety of the drug. VAS was used to measure pain severity. Secondary to pain, the patients' health-related quality of life (HRQoL) was also assessed using the Muscle and Joint Measure (MJM) scale. Safety was monitored on the first dose by the patients. These assessments were done at each study visit.

Description of study visits and timing of assessments

Entry Visit (V1) (Baseline)

After signing informed consent, the patients were evaluated, and selection was made based on inclusion/exclusion criteria. Patient demographics, vital signs (including body temperature, respiratory rate, blood pressure), relevant medical history, systemic examination, VAS scale scoring for pain, MJM scale for QoL, concomitant medications, and study medication was recorded in the case report form (CRF).

Follow-Up Visit (V2)

A follow-up visit was planned for all enrolled patients one to two weeks after the baseline visit. These subjects were evaluated for the clinical response of the study drug and pain status, QoL was re-recorded in the follow-up visit, and overall effectiveness and safety were assessed. The drug safety was determined based on the frequency of adverse event(s) (AEs), evaluated through the AE form provided within the CRF.

Ethics approval and consent to participate

Ethical approval was obtained from Pakistan Medical Association Committee on Ethics (Reference no: OS/908/PWQ/10). The patients were enrolled after signing informed consent.

Statistical analysis

Data analysis was done using Statistical Package for Social Sciences (SPSS) version 21.0 (IBM Corp., Armonk, NY). All categorical variables were displayed using frequency (percentages) while all continuous variables were given as mean ± SD. Chi-square and T-test were used to compare the pre and post-treatment qualitative and quantitative values respectively. P-value ≤ 0.05 was taken as significant.

## Results

In this multicenter study, the mean age of the included patients was 47.24 ± 14.20 years. The male to female ratio was nearly equal. Moreover, 62% of patients had normal BMI; vitals were also stable in most cases. The majority of the enrolled cases were non-smokers (89.7%) and non-alcoholics (99.7%). Further details regarding the baseline characteristics of the study participants are given in Table 1.

**Table 1:** Presentation of the general baseline information

Variable		N=399
Age; years (Mean±SD)		47.24±14.20
Gender	Male	191(49.4)
	Female	194(50.6)
BMI; kg/m^2 ^	Underweight	7(1.7)
	Normal	240(60.1)
	Overweight	62(15.5)
	Obese	84(21)
	Very Obese	6(1.7)
Respiratory rate; breaths per min (Mean±SD)		23.29±18.50
Heart Rate; beats per min (Mean±SD)		75.66±6.3
Body Temperature; ºF (Mean±SD)		98.7±1.42
Smoking	No	358(89.7)
	Yes	41(10.2)
Alcohol Intake	Yes	1(0.3)
	No	398(99.7)
Comorbidities	None	278(69.6)
	Hypertension	59(14.7)
	Diabetes Mellitus	26(6.5)
	Diabetes Mellitus + hypertension	32(8)
	Epilepsy	1(0.25)
	Gout	1(0.25)
	Asthma	1(0.25)

Most of the enrolled participants had knee osteoarthritis (OA) (38.4%) followed by backache (18.2%), rheumatoid arthritis (RA; 7.5), generalized muscular pain (7.3%), and frozen shoulder (6.0%) while other musculoskeletal conditions or pain conditions are mentioned in Table 2.

**Table 2:** Frequency of musculoskeletal disorders

Musculoskeletal Disorder	N(%)
Knee Osteoarthritis	148(38.4)
Backache	70(18.2)
Frozen Shoulder	23(6.0)
Rheumatoid Arthritis	29(7.5)
Generalized Muscular Pain	28(7.3)
Soft Tissue Injury	22(5.7)
Post-Operative Muscular Pain	6(1.6)
Toothache	14(3.6)
Sciatic Nerve Compression	9(2.3)
Others (Septic Arthritis, Gaucher Disease, Tension Headache, Hip OA, etc.)	36(9.4)

**Figure 1:**
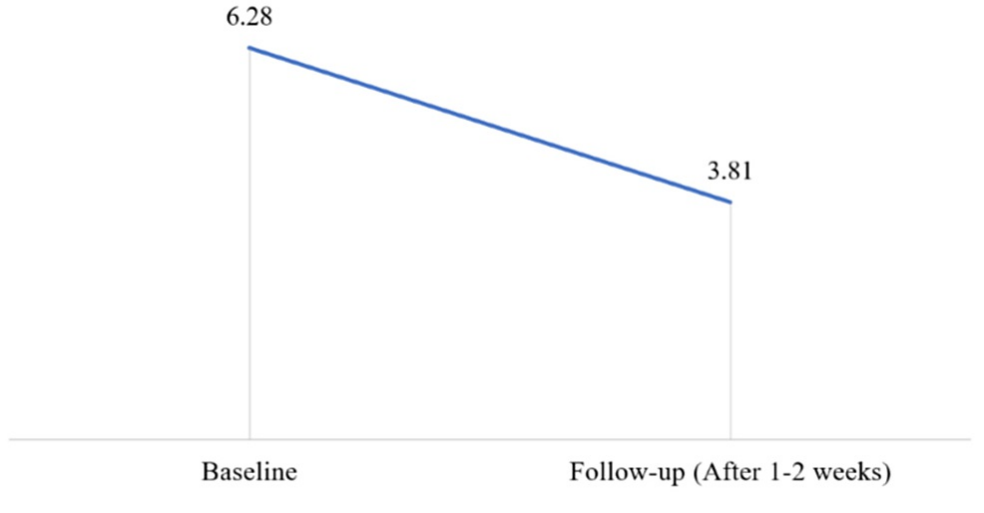
Mean reduction in visual analog scale (VAS) pain scores from baseline to the follow-up visit.

A significant decrease was observed in the pain severity after the treatment with Nuberol Forte (paracetamol 650 mg + orphenadrine 50 mg) (p<0.05), as shown in Figure 1, i.e., 6.18 ± 1.83 (VAS baseline) vs. 3.70 ± 2.22 (VAS follow-up).

At the baseline visit, 24 patients had mild pain, mainly OA (45.5%), followed by frozen shoulder, soft tissue injury, RA, backache, and generalized muscular pain. One hundred ninety-one had moderate pain, mainly with osteoarthritis (51.3%), backache (18.1%), frozen shoulder (7.3%), RA (6.2%), and others, including sciatic nerve compression, soft tissue injury, neck pain, dysmenorrhea, toothache, and operative pain. Furthermore, 164 patients complaint of the severe form of the pain, of which 29.2% was due to osteoarthritis followed by backache (18.9%), rheumatoid arthritis (9.1%), toothache (7.3%), and the remaining had frozen shoulder, dysmenorrhea, soft tissue injury, earache, and sciatic nerve compression. While on the follow-up visit, only 29 patients had severe pain.

There was a significant change in the pain grade before and after treatment among patients with various musculoskeletal disorders. In case of severe and worst pain, Nuberol Forte was prescribed with diclofenac sodium or aceclofenac. See Table 3.

**Table 3:** Types of musculoskeletal disorders in relation to improvement in the pain grade from baseline to the follow-up visit *p-value<0.05 is considered statistically significant.

Musculoskeletal Disorder	Pain Grade Baseline	Pain Grade Follow-Up					p-value
		No pain	Mild	Moderate	Severe	Worst pain	
Knee OA	Mild	2(25)	6(75)	-	-	-	0.000*
	Moderate	-	61(68.5)	28(31.5)	-	-	
	Severe	-	9(18)	28(56)	13(26)	-	
	Worst Pain	-	-	-	-	1(100)	
Backache	Mild	-	4(100)	-	-	-	0.002*
	Moderate	1(2.9)	27(77.1)	7(20)	-	-	
	Severe	-	12(40)	13(43.3)	5(16.7)	-	
	Worst Pain	-	-	-	1(100)	-	
Frozen Shoulder	Mild	-	3(100)	-	-	-	0.448
	Moderate	-	4(66.7)	2(33.3)	-	-	
	Severe	-	6(46.2)	5(38.5)		-	
	Worst Pain	-	-	1(100)	-	-	
RA	Mild	-	2(100)	-	-	-	0.560
	Moderate	-	10(83.3)	2(16.7)	-	-	
	Severe	-	9(60)	5(33.3)	1(6.7)	-	
	Worst Pain	-	-	-	-	-	
Generalized Muscular Pain	Mild	-	1(100)	-	-	-	0.020*
	Moderate	-	6(75)	2(25)	-	-	
	Severe	-	3(15.8)	11(57.9)	5(26.3)	-	
	Worst Pain	-	-	-	-	-	
Soft Tissue Injury	Mild	-	3(100)	-	-	-	0.153
	Moderate	-	12(85.7)	2(14.3)	-	-	
	Severe	-	1(25)	2(50)	1(25)	-	
	Worst Pain	-	-	-	-	-	
Post-OP Muscular pain	Mild	-	-	-	-	-	0.540
	Moderate	-	-	2(100)	-	-	
	Severe	-	1(33.3)	2(66.7)		-	
	Worst Pain	-	-	1(100)	-	-	
Toothache	Mild	-	-	-	-	-	0.180
	Moderate	-	2(100)	-	-	-	
	Severe	-	6(50)	6(50)	-	-	
	Worst Pain	-	-	-	-	-	
Sciatic Nerve Pain	Mild	-	-	-	-	-	0.570
	Moderate	-	5(71.4)	2(28.6)	-	-	
	Severe	-	1(50)	1(50)	-	-	
	Worst Pain	-	-	-	-	-	
Others	Mild	-	3(100)	-	-	-	0.490
	Moderate	-	12(75)	4(25)	-	-	
	Severe	-	10(62.5)	5(31.3)	-	-	
	Worst Pain	-	-	1(100)	-	-	

The results of the MJM (QoL) assessment showed that the mean score for severity of muscle cramps or spasms reduced from 7.07 ± 1.54 (baseline) to 4.75 ± 2.20 (follow-up) (Table 4). There was no significant difference in the emotional impact of the muscle spasm pre and post-treatment. Medication has resulted in improved physical activity, sitting, and social activity at a significant level (p=0.01). In the 'Muscle weakness section,' the mean severity reduced from 6.32 ± 1.93 to 4.0 ± 2.26 (p=0.01). Similarly, myalgia and arthralgia also significantly improved, as observed via the severity rating on the follow-up visit. It was noted that there was an overall improvement in the quality of life of the enrolled patients with musculoskeletal disorders after treatment with Nuberol Forte.

**Table 4:** Comparison of the pre and post-treatment quality of life using the MJM questionnaire *p-value<0.05 is considered statistically significant. MJM: Muscle and Joint Measure

MJM Questions				Baseline	Follow-up visit	p-value
Muscle Cramps or Spasms						
Rate severity of cramps at their worst (0-10)				7.07±1.54	4.75±2.20	0.01*
When do you have muscle cramps or spasms?				2.97±1.39	1.68±1.31	0.01*
How much time do you have muscle cramps or spasms in a usual month?				6.46±2.35	3.97±2.47	0.01*
How severe are your muscle cramps or spasms on				6.74±1.49	4.43±2.04	0.01*
Do cramps or spasms wake you when you are sleeping or make it difficult to get to sleep?				1.92±1.17	0.94±1.07	0.01*
1d2. How much do cramps or spasms impact your emotional well-being?				1.84±0.79	1.08±0.86	0.56
How much do muscle cramps or spasms limit or prevent each of these activities?	Physical activities like sports or exercise			2.12±0.78	1.33±0.99	0.01*
	Sitting or standing			1.83±0.83	0.91±0.98	0.01*
	Social activity			1.42±0.75	0.66±0.80	0.01*
Muscle Weakness						
Severity of muscle weakness at their worst (0-10)				6.32±1.93	4±2.26	0.01*
Has a doctor told you that you have muscle weakness related to a “neuropathy”?				0.35±0.73	0.29±0.58	0.01*
How much of the time do you have muscle weakness in a usual month?				9±0.13	5.02±2.08	0.01*
How severe is your muscle weakness?				6.27±1.95	3.79±2.11	0.01*
Does muscle weakness make you need to take naps or sleep longer?				1.54±1.23	0.73±0.89	0.01*
How much does muscle weakness impact your emotional well-being?				1.51±1.04	0.85±0.84	0.01*
How much does muscle weakness limit or prevent each of these activities?		Physical activities like sports or exercise		2±0.74	1.17±0.96	0.01*
		Sitting or standing		1.62±0.89	0.79±0.91	0.01*
		Social activity		1.78±0.67	0.97±0.83	0.01*
Myalgias						
Severity of aches, pains, stiffness, or other muscle problems at their worst (0-10)				6.30±1.69	3.77±1.89	0.01*
How much of the time do you have these Muscle aches, pains, stiffness, or other muscle problems in a usual month?				7.18±1.95	4.75±2.23	0.01*
How severe your muscle problems are on the 0 to 10 scale. Select the number that best fits how severe your aches, pains, stiffness, or other muscle problems are usually or most of the time:				6.26±1.69	3.79±1.89	0.01*
Do muscle aches or pains, stiffness, or other problems wake you when you are sleeping or make it difficult to get to sleep?				1.75±1.04	0.86±0.94	0.01*
How much do muscle aches or pains, stiffness, or other problems impact your emotional well-being?				1.48±1.01	0.74±0.86	0.01*
How much do muscle aches, pains, stiffness, or other problems limit or prevent each of these activities?			Physical activities like sports or exercise	1.97±0.66	1.14±0.83	0.01*
			Sitting or standing	1.58±0.69	0.81±0.73	0.01*
			Social activity	1.79±0.64	0.93±0.75	0.01*
Arthralgias						
Severity of joint or spine problems at their worst (0-10)				6.21±1.90	3.81±2.05	0.01*
Where do you have joint or spine problems?				1.04±0.60	1.03±0.34	0.01*
How much of the time do you have joint or spine problems in a usual month?				7.14±2.12	4.97±2.28	0.01*
How much difficulty do you have, or how limited are you, when moving your joints or spine?				2.24±0.69	1.47±0.93	0.01*
How severe your joint or spine problems are on the 0 to 10 scale by selecting the number that best fits how severe your problems are USUALLY or most of the time				6.13±1.81	3.74±1.93	0.01*
Do joint or spine problems wake you when you are sleeping or make it difficult to get to sleep?				1.58±1.10	0.88±1.05	0.01*
How much do joint or spine problems impact your emotional well-being?				1.36±0.95	0.77±0.86	0.01*
How much do joint or spine problems limit or prevent each of these activities?			Physical activities like sports or exercise	1.89±0.79	1.17±0.83	0.01*
			Sitting or standing	1.53±1.40	0.84±0.77	0.01*
			Walking	1.43±0.77	0.78±0.72	0.01*
			Social activity	1.65±0.80	0.93±0.76	0.01*
Bone or Spine Fractures						
Have you had any bone or Spine fractures (broken bones) since your treatment?			No	298(90.6)	287(87.2)	0.39
			Yes	30(9.1)	32(9.7)	
How many fractures have you had since your treatment?			None	8(2.4)	283(86.0)	0.001*
			One	29(8.8)	28(8.5)	
			Two or more	3(0.9)	2(0.6)	

During the study, only 10 patients reported mild AEs; three of these were adverse drug reactions (ADRs) associated with Nuberol Forte and seven were reported by the patients treated with Nuberol Forte and other NSAIDs. Dryness of the mouth (n=2), dizziness (n=1), gastric irritation (n=3), tachycardia (n=1), restlessness (n=1), Palpitation (n=1), and itching (n=1) were the AEs observed.

## Discussion

This study provides real-life evidence on the effectiveness, safety, and tolerability of the paracetamol with orphenadrine combination (Nuberol Forte) for musculoskeletal pain in Pakistani practice. Internationally, multiple studies have addressed the role of the paracetamol with orphenadrine combination in pain management in different musculoskeletal conditions [21-25], but there is still a lack of local literature. The small number of well-controlled studies on humans supports that the combination has superior efficacy over paracetamol alone [22-23]. Only one local study by Sajid et al. investigated the role of the orphenadrine + paracetamol combination in fever and myalgia in local viral infections. The study results showed the effectiveness in controlling fever and managing myalgia with a better safety profile [20].

Hunskaar et al.'s study on mice showed increased and prolong antinociceptive effects of paracetamol in mice, and a combination of the paracetamol with orphenadrine showed a significantly improved effect as compared to either of the drugs alone [22-23]. Similarly, a review article also supported that the combination has more efficacious impacts than placebo or paracetamol alone [21]. Tervo et al. conducted a study on back pain and its treatment with combination therapy [24]. They found that combination was more effective than paracetamol in patients with low back pain [24]. In our study, 70 subjects had complaints of backache; a positive response was observed post-treatment with the provided combination therapy and significantly improved quality of life during the pain management process. Winter et al., in their study on oral post-surgical pain and the effects of analgesic combinations with orphenadrine [25], showed that the patients with postoperative oral surgical pain (moderate to severe) responded well to a combination of orphenadrine-paracetamol [25].

Furthermore, 14 patients in the current study had a toothache; all of them responded well with the provided combination regime and reflected an overall improvement in the quality of life. The McGuinness trial was designed to compare the pain management response of the combination (orphenadrine citrate and paracetamol) and paracetamol alone [26]. The study measured pain, stiffness, and functional impairment in patients with musculoskeletal disorders; the results indicated a significant difference with the combination and betterment in all three parameters. Our study was designed similarly to the McGuinness BW trial, assessing the pain with the VAS scale, muscle weakness, joint, and spine with the MJM scale. In our study, significant improvement was observed in pain severity and quality of life was observed after treatment with Nuberol Forte.

Concerning the association between lifestyle and musculoskeletal pain, a number of studies identified various risk factors in association with musculoskeletal pain. Walsh et al. conducted a systematic review and meta-analysis to assess the association between body fat and musculoskeletal pain [27]. The results identified the positive association between increased body mass and widespread single-site joint pain in the knee, foot, and lower back [28]. Moreover, the association between smoking and musculoskeletal pain has also been investigated in previous studies. A meta-analysis revealed a strong association between smoking and low back pain among adolescents [29]. Among other identifiable risk factors is alcohol consumption; a systematic review including nine observational studies concluded no association of alcohol consumption with low back pain [30]. Whereas a study also displayed a negative association between alcohol consumption and osteoarthritis [31]. Diabetes mellitus is another considered risk factor for musculoskeletal pain. The study by Pai et al. showed that type 2 diabetes patients with age between 18 and 50 years have a higher 10-year cumulative incidence of musculoskeletal pain than the non-diabetic group [32]. However, most of our patients had normal BMI (60.1%), were non-smokers (89.7%), non-alcoholics (99.7%), and had no other comorbid condition (69.6%). Hence, the rest with comorbidities or risk factors other than those listed above might have promoted musculoskeletal pain that requires further investigation.

Globally, NSAIDs are usually prescribed analgesics although gastrointestinal (GI) complications are well-established. The associated drug adverse effects with traditional NSAIDs, cyclooxygenase-2 inhibitors (COX-2) inhibitors, and acetylsalicylic acid (ASA) [28,33] have left physicians confused while selecting the best regime for pain management, especially for the use of analgesics for a longer period and in special patient populations. Paracetamol is the most widely used nonprescription pain medication, is likely effective, well-tolerated, and has a good safety profile [34]. The safety of paracetamol has been established through clinical trials, systematic reviews, meta-analyses, and epidemiologic studies except for the reported adverse effects of acute renal failure (ARF) and acute liver failure associated with overdoses of paracetamol [34]. Therefore, a cautiously considered treatment strategy is required to avoid the dose-limiting adverse events of single-agent therapy. The combination of lower doses of multiple analgesics is an attractive approach for maximizing the benefit-risk ratio of pharmacotherapy for pain. Paracetamol is the first-line agent for managing mild-to-moderate pain, and its combination with analgesics is widely prescribed. The paracetamol combination with orphenadrine (Nuberol Forte) is prescribed alone or with other analgesics (aceclofenac 200 mg and diclofenac sodium 50 mg), especially in the moderate and severe pain in different musculoskeletal conditions by the investigators in their routine practice. A significant difference was noted in the mean pain score before and after the treatment (p-value<0.05) in all the reported musculoskeletal conditions. In the study, the combination was well-tolerated and fewer adverse effects were observed that were mild in nature.

Musculoskeletal pain is the foremost problem in the psychosocial and physical wellbeing of an individual and significantly impairs the subject’s quality of life [11]. The current study also investigated the quality of life of the local population with musculoskeletal pain. Globally, it is recognized that chronic pain harms the human quality of life. The impact of pain affects the general health with the social and psychological well-being of the human. In this study, the Muscle and Joint Measure (MJM) scale was selected to address the key areas of health-related quality of life of patients with different musculoskeletal conditions who were managed with tab Nuberol Forte (paracetamol and orphenadrine) in the Pakistani population. In the study, the specific medication has resulted in the improvement of physical activity, sitting, and social activity at a significant level (p<0.05).

Limitation

One of the significant limitations of the study was the limited sample size. Moreover, our objective was limited to safety and quality of life. Longitudinal, large-scale, multicenter studies should be conducted focusing on psychosocial and physical well-being in the future.

## Conclusions

The paracetamol and orphenadrine combination (Nuberol Forte) exhibited a positive clinical response to musculoskeletal pain and improved patients' quality of life. Overall, Nuberol Forte is a well-tolerated treatment choice with fewer adverse effects than NSAIDS or other therapeutic agents.
